# General Medicine and Therapeutics

**Published:** 1896-01

**Authors:** 


### *SELECTIONS AND ABSTRACTS.*

### GENERAL MEDICINE AND THERAPEUTICS.

Edited by Dr. Luther B. Grandy.

The Treatment of Migrain. (Dr. W. O. Hermance, *Philadelphia Polyclinic.*)

For the permanent cure of hemicrania various remedies have been proposed. Some of these, if persistently followed out, with attention to the diet and management of the lithemia which is manifest in so many cases, will result in ultimate cure. To the practical mind, however, it will be of most interest to know what means have been used for the paroxysms when the patient demands immediate ease from his sufferings.

Among the drugs useful for this purpose antipyrin ranks among the foremost. It should be given in rather large doses, say 10 to 15 grains repeated in an hour. Given early it often aborts the attack. Many prefer to give moderate doses of digitalis with the antipyrin to offset its well-known depressing action upon the heart.

Caffein has been found useful in migrain. Either the citrate or the pure alkaloid may be used, with the preference given to the former preparation. This may be exhibited in divided doses of ½ grain each, every ten or fifteen minutes, until relief is obtained, or as is preferred by H. C. Wood, in a single dose of 5 grains given at the outset of pain.

Phenacetin has been used largely, and has been found very useful in doses of 7 grains repeated twice in as many hours.

Acetanilid acts like its companions of the coal-tar group, and is often found useful when combined with caffein and monobromated camphor, according to the formula of Dr. S. Solis-Cohen, as follows:

| | |
|---|---|
| Acetanilid | 3 grains. |
| Monobromated camphor | 3 grains. |
| Caffein | 2 grains. |

Mix.—Give every hour until headache is relieved, or three powders have been taken.

Dr. Cohen sometimes gives acetanilid in solution with fluid extract of guarana (30 minims) in place of caffein. An elixir of guarana may be made quite pleasant to the taste.

Ammonium chlorid has been recommended. It can be given in 30 grain doses either with the tincture of aconite root (2 to 4 drops) or alone.

Amyl nitrite has often been found to be useful. Given by inhalation it sometimes subdues the most stubborn attack, but usually upon its discontinuance the pain will recur.

Crystallized hyoscin given hypodermically in doses from $\frac{1}{40}$ to $\frac{1}{60}$ of a grain will often afford relief for several hours.

The bromids and chloral may be given either in conjunction or separately, due attention, of course, being given to their depressant qualities.

Moderate doses of strychnine, either by the mouth or under the skin, will sometimes succeed when everything else fails, the drug acting by raising the standard of nutrition of the part.

The use of external applications is very often fruitless. Many have tried local heat, cold, aconite, menthol, and galvanism. Sometimes, however, they may assist in the result, and give temporary comfort until the drug used internally can make its power manifest.

Opium and its preparations have been left until the last, because they should not be used. The reason is apparent. While relief is sure, the attack is bound to recur, and the patient will demand morphine, and by yielding to his request for the drug, the physician exposes his patient to the greater danger of the dreaded morphine habit.

When the attack becomes fully developed, all that can be done after trying the various remedial measures, is to place the patient in a perfectly dark room, and surround him with absolute quiet.

Finally the physician may reassure his patient that there is a time coming when the disease will cease spontaneously, and by following up the interparoxysmal treatment, and the careful use of the remedies mentioned above, can make his patient's life quite comfortable.

### ALBUMINURIA AND LIFE INSURANCE.

After an interesting paper on this subject in the *Medical Examiner* (October), Dr. R. L. Faithful concludes:

1. As the clinical significance of albumen in the urine of otherwise apparently healthy individuals, has not yet been finally or satisfactorily determined by the majority of the most careful observers, it must not be expected that life insurance companies will accept such risks, except reluctantly, and then only after most careful and repeated chemical and microscopical examinations of the urine by known and trusted medical examiners.

2. Albuminuria must be viewed with suspicion and distrust. That of a persistent character, from whatever cause, calls for rejection.

3. Albuminuria in applicants under forty years of age, who present a good family and personal history, who are of good physique, and of good habits, and are free from any taint, hereditary or acquired, the amount of albumen being small and transient, may after careful and repeated examinations of the urine, be accepted on short endowment plans of insurance, or upon other plans, with an extra premium to cover substantial loading.

4. Albuminuria dependent upon or following la grippe, and not present three to six weeks after in otherwise healthy applicants, the specific gravity of whose urine for the twenty-four hours is not below 1020, may be accepted on ordinary life policies, without loading.

5. Any applicant with albumen in the urine, with increased arterial tension, with urine of low specific gravity, with any indications of hypertrophy of the heart or accentuation of the second aortic sound, should be rejected.

6. Any applicant with a persistently low specific gravity, with or without albumen in the urine, should be rejected, and more especially so if with the above he suffers from occasional occipital headaches.

7. In all cases of albuminuria the condition or state of the arteries should be carefully investigated, especially so, if the applicant is over forty.

